# Supplementary material for: Remodelling of the Mitochondrial Bioenergetic Pathways in Human Cultured Fibroblasts with Carbohydrates
Source: Biology (Basel). 2023 Jul 14;12(7):1002. doi: 10.3390/biology12071002 (PMC10376623; doi:10.3390/biology12071002)
Supplement: Supplementary file 1 [file biology-12-01002-s001.zip › Biology Taanman/Figure S1.pdf]

β-actin, 1:120,000

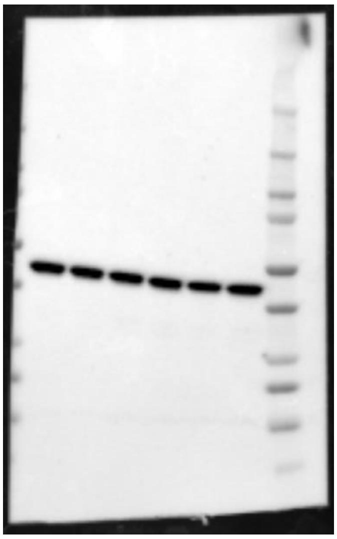

MTCO2, 1:1,000

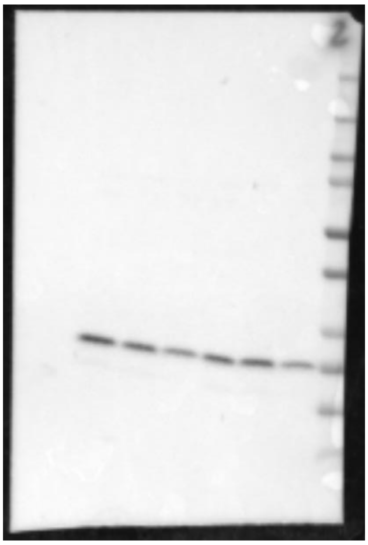

HK1, 1:1,000

(Unrelated protein)

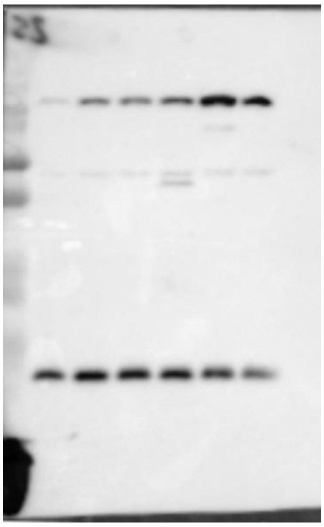

ATP5A, 1:3,000
(SOD2)

TFAM, 1:1,000

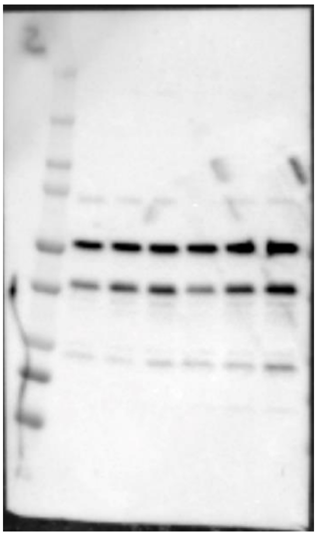

UQCRC2, 1:3,000
(SOD2)

NDUFB6, 1:2,000

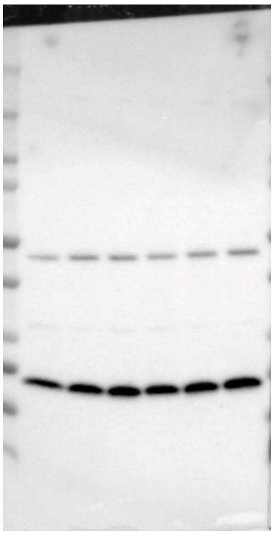

MCT4, 1:1,000

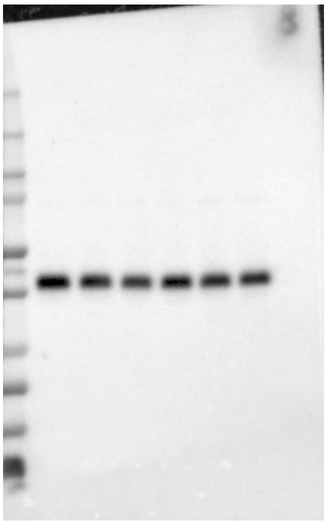

COX4, 1:1,000

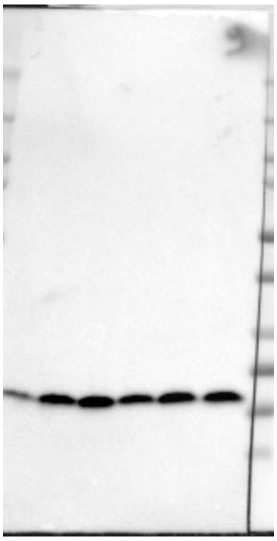

pPDHA (S293), 1:100,000

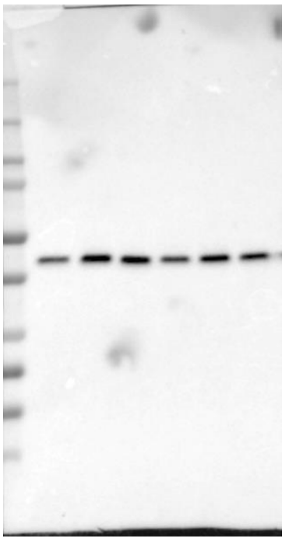

MTCO1, 1:1,000

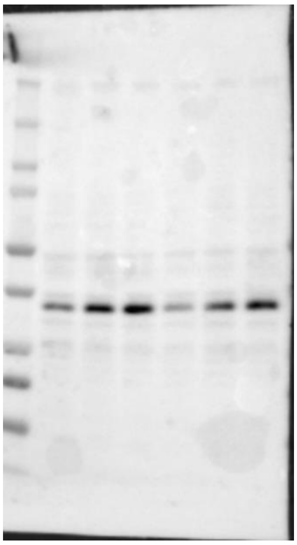

GAPDH, 1:120,000

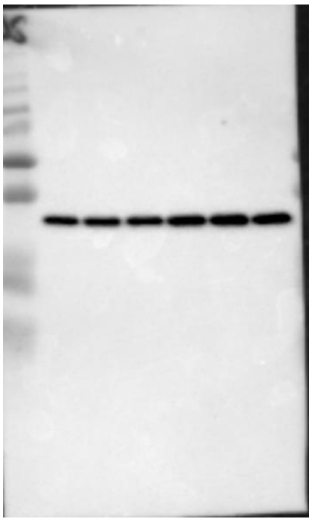

TOMM20, 1:750

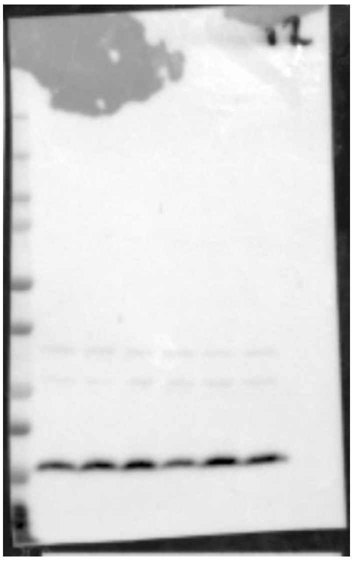

SDHA, 1:2,000

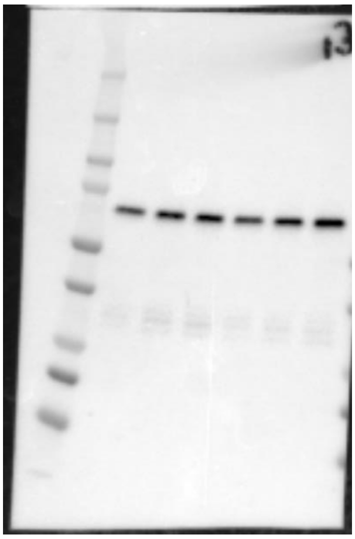

PKM2, 1:1,000

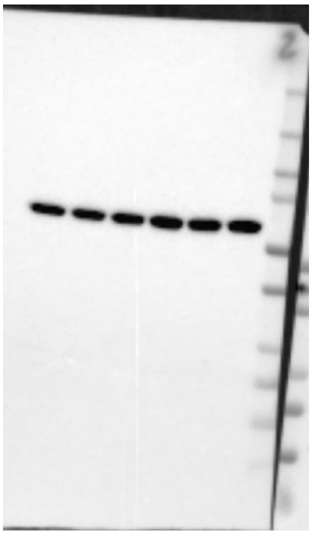

TFAM, 1:1000

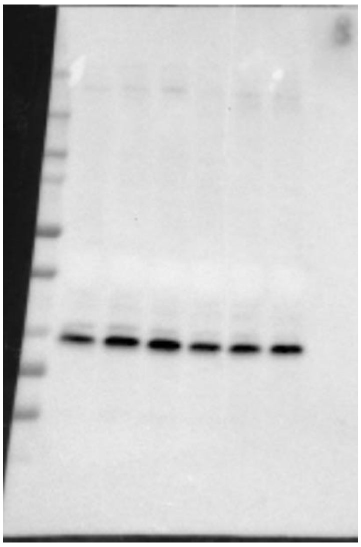

Figure S1. Uncropped western blot images.
