# Supplementary material for: Remodelling of the Mitochondrial Bioenergetic Pathways in Human Cultured Fibroblasts with Carbohydrates
Source: Biology (Basel). 2023 Jul 14;12(7):1002. doi: 10.3390/biology12071002 (PMC10376623; doi:10.3390/biology12071002)
Supplement: Supplementary file 1 [file biology-12-01002-s001.zip › Biology Taanman/Table S1.pdf]

Table S1. Primers used for ddPCR

Target	Forward primer	Reverse primer
mtDNA D-loop	5’-CATCTGGTTCCTACTTCAGGG	5’-TGAGTGGTTAATAGGGTGATAGA
B2M (nuclear DNA)	5’-TGCTGTCTCCATGTTTGATGTATCT	5’-TCTCTGCTCCCCACCTCTAAGT
**Target**	**TaqMan Probe**	
mtDNA D-loop	6FAM-CTTAAATAAGACATCACGATGGATCAC-TAMRA	
B2M (nuclear DNA)	VIC-TTGCTCCACAGGTAGCTCTAGGAGG-TAMRA	
