# Supplementary material for: Remodelling of the Mitochondrial Bioenergetic Pathways in Human Cultured Fibroblasts with Carbohydrates
Source: Biology (Basel). 2023 Jul 14;12(7):1002. doi: 10.3390/biology12071002 (PMC10376623; doi:10.3390/biology12071002)
Supplement: Supplementary file 1 [file biology-12-01002-s001.zip › Biology Taanman/Table S2.pdf]

Table S2. Primers used for RT qPCR.

Target	Forward primer	Reverse primer
Designed with Primer-BLAST		
ACTB (β-actin gene)	5'-GGACTTCGAGCAAGAGATGG	5'-AGCACTGTGTTGGCGTACAG
MTCO2	5'-CATGCAGCGCAAGTAGGCTC	5'-CGGGCAGGATAGTTCAGACG
MTND6	5'-GGTGCTGTGGGTGAAAGAGT	5'-CTCCCGAATCAACCCTGACC
MTRNR2 (16S rRNA gene)	5'-GGTGCAGCCGCTATTAAAGG	5'-ATCATTTACGGGGGAAGGCG

Target	Catalogue Number
Purchased from Qiagen	
COX4I1	QT00065961
GAPDH	QT00079247
NDUFB7	QT00094129
NDUFV2	QT00077630
NDUFB7	QT00094129
NRF1	NM_005011
PPARGC1A (PGC-1α gene)	QT00095578
TOMM20	QT00088914
