# Supplementary material for: Remodelling of the Mitochondrial Bioenergetic Pathways in Human Cultured Fibroblasts with Carbohydrates
Source: Biology (Basel). 2023 Jul 14;12(7):1002. doi: 10.3390/biology12071002 (PMC10376623; doi:10.3390/biology12071002)
Supplement: Supplementary file 1 [file biology-12-01002-s001.zip › Biology Taanman/Table S3.pdf]

Table S3. Primary and secondary antibodies used for western blot analyses.

Target	Type	Supplier	Catalogue Number
Primary antibodies			
ATPA5	mouse mAb	Abcam	ab14748
COX4	mouse mAb	Abcam	ab110261
GAPDH	mouse mAb	Abcam	ab8245
HK1	rabbit mAb	Abcam	ab150423
MCT4	rabbit pAb	ProteinTech	22787-1-AP
MTATP6	mouse mAb	Abcam	ab219825
MTCO1	mouse mAb	Abcam	ab14705
MTCO2	mouse mAb	Abcam	ab110258
MTCYB	mouse mAb	Abcam	ab219823
NDUFB8	mouse mAb	Abcam	ab110242
pPDHA1 (S293)	rabbit mAb	Abcam	ab177461
PKM2	rabbit mAb	Cell Signaling Technology	4053S
SDHA	mouse mAb	Abcam	ab14715
TFAM	mouse mAb	ThermoFisher Scientific	MA5-16148
TOMM20	rabbit pAb	Santa Cruz Biotechnology	sc-11415
UQCRC2	mouse mAb	Abcam	ab14745
β-actin	mouse mAb	Abcam	ab6276
Secondary antibodies			
Mouse IgG	goat pAb	Agilent Technologies/ Dako	P0447
Rabbit IgG	goat pAb	Agilent Technologies/Dako	P0448

Abbreviations: mAb, monoclonal antibody; pAb, polyclonal antibody
